# Exploring the Potential of a Highly Scalable Metal-Organic Framework CALF-20 for Selective Gas Adsorption at Low Pressure

**DOI:** 10.3390/polym15030760

**Published:** 2023-02-02

**Authors:** Mostafa Yousefzadeh Borzehandani, Majid Namayandeh Jorabchi, Emilia Abdulmalek, Mohd Basyaruddin Abdul Rahman, Muhammad Alif Mohammad Latif

**Affiliations:** 1 Integrated Chemical BioPhysics Research, Faculty of Science, Universiti Putra Malaysia, Serdang 43400, Selangor, Malaysia; 2 Foundry of Reticular Materials for Sustainability, Institute of Nanoscience and Nanotechnology, Universiti Putra Malaysia, Serdang 43400, Selangor, Malaysia; 3 Leibniz Institute for Catalysis, Albert-Einstein-Straße 29a, D-18059 Rostock, Germany; 4 Centre of Foundation Studies for Agricultural Science, Universiti Putra Malaysia, Serdang 43400, Selangor, Malaysia

**Keywords:** metal-organic framework, CALF-20, selective adsorption, grand canonical monte carlo, molecular dynamics

## Abstract

In this study, the ability of the highly scalable metal-organic framework (MOF) CALF-20 to adsorb polar and non-polar gases at low pressure was investigated using grand canonical Monte Carlo (GCMC) and molecular dynamics (MD) simulations. The results from the simulated adsorption isotherms revealed that the highest loading was achieved for SO_2_ and Cl_2_, while the lowest loading was found for F_2_ molecules. The analysis of interaction energies indicated that SO_2_ molecules were able to form the strongest adsorbent-adsorbate interactions and had a tight molecular packing due to their polarity and angular structure. Additionally, Cl_2_ gas was found to be highly adsorbed due to its large van der Waals surface and strong chemical affinity in CALF-20 pores. MD simulations showed that SO_2_ and Cl_2_ had the lowest mobility inside CALF-20 pores. The values of the Henry coefficient and isosteric heat of adsorption confirmed that CALF-20 could selectively adsorb SO_2_ and Cl_2_. Based on the results, it was concluded that CALF-20 is a suitable adsorbent for SO_2_ and Cl_2_ but not for F_2_. This research emphasizes the importance of molecular size, geometry, and polarity in determining the suitability of a porous material as an adsorbent for specific adsorbates.

## 1. Introduction

Metal-organic frameworks (MOFs) are a sub-class of porous materials with one-, two-, and three-dimensional networks that are constructed by metal ions/clusters and organic linkers [1]. MOFs have shown advantageous features when compared to conventional porous materials; (i) experimental measurement of BET (Brunauer, Emmett and Teller) for zirconium-based MOF, NU-1103, recorded an ultra-high surface area (6550 m^2^/g) [2], (ii) variable-temperature powder x-ray diffraction (VT-PXRD) and thermal gravimetric (TG) analyses for Co-based MOF, NJU-Bai62 indicated surprising thermal stability of up to 450 °C [3], (iii) D-glucose selective hydrogenation reaction tests using ruthenium-impregnated, chromium-based MIL-100 exhibited an exceptional catalytic activity, and recyclability up to 12 runs [4], and (iv) revised auto-correlations (RACs) analysis using the machine learning method has recently updated that there are more than 90,000 synthesized and 500,000 predicted MOF structures, highlighting MOFs’ high structural diversity [5].

Although MOFs have been considered for energy conversion [6], water treatment [7], catalysis [8], photodynamic therapy [9], and drug delivery [10], their applications for gas adsorption and separation have been extensively studied [11,12,13,14,15,16,17]. This can be addressed by the features and characteristics that make MOFs an ideal porous material for research and practical use for gas adsorption and separation. For example, Jian-Rong Li and colleagues designed an organic linker that resulted in two interpenetrated frameworks, BUT-43 and BUT-44, after mixing with paddlewheel Cu_2_(COO)_4_ and Zr_6_O_4_(OH)_8_(COO)_8_ clusters, respectively [18]. Adsorption isotherm showed that both frameworks had the highest uptake and selectivity for C_2_H_2_ over CO_2_ and CH_4_ due to better chemical affinity and surface contact. Moreover, combining pyridine-based acylamide-linking diisophthalate with dicopper(II)-paddlewheel clusters produced the acylamide-functionalized MOF, HNUST-8 [19]. It was found that this framework had high selectivity for CO_2_ over CH_4_ and N_2_ caused by its strong Lewis acid-base interactions with open metal site Cu(II) and hydrogen interactions with acylamide functional groups (-CONH … OCO). In addition, it was shown that developing the polarity of pore surface using O- and N-rich organic linkers via the pre-functionalization process could improve the adsorption of gas molecules. For instance, titanium-based MOF, NTU-9, demonstrated a one-dimensional channel containing polar oxygen atoms, and it was capable of excellent C_2_H_2_ adsorption [20]. Surface area, chemical affinity, presence of polar/non-polar functional groups, and polarity of pore surface are the most determinative factors for gas adsorption. 

**Figure 1:**
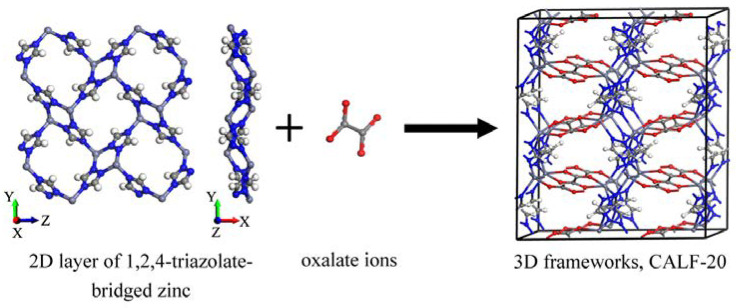
Schematic diagram for the synthesis of CALF-20.

Regardless of MOFs being an ideal platform for gas adsorption, they must be produced on a large scale for industrial applications [21]. Recently, Shimizu et al. have synthesized a low-priced and scalable zinc-based MOF, CALF-20 (Calgary Framework 20) [22]. More than 35% of dried solid CALF-20 was extracted per total amount of solvents used, plus an extraordinary space-time yield (STY) for the precipitation step of 550 kg/m^3^ per day. In comparison with this achievement, it is worth noting that the STYs for zeolites are observed in the range of 50 to 150 kg/m^3^ per day [23]. The 3D frameworks were built by first synthesizing 2D layers of 1,2,4-triazolate-bridged zinc(II) ions, which were subsequently pillared by oxalate ions (Figure 1). The reactants are cost-effective, commercially available, and environmentally safe because the reaction can be accomplished in a water/methanol mixture (less than 25 wt% organic solvents) [22]. More attention should be paid to CALF-20 MOF since it can be synthesized using a sustainable methodology. This can be emphasized as scalability and cost-efficiency are the major parameters to consider when transitioning from academia to commercialization [21]. Concerning the environmental impacts of hazardous organic linkers, metal complexes, and solvents, focusing on green synthesized MOFs such as CALF-20 guarantees clean MOF-based technologies [24]. In terms of CALF-20 applications in gas adsorption, it demonstrated a high capacity for CO_2_ (up to 5.0 mmol⋅g^−1^ at 1.2 bar and 273 K), as well as outstanding selectivity of CO_2_ over water (below 40% relative humidity). In addition, for a 10:90 CO_2_/N_2_ mixture, the estimated selectivity of CO_2_ over N_2_ using the ideal adsorbed solution theory (IAST) was 230. CALF-20 was tested for the separation of CO_2_ from flue gas (containing CO_2_/N_2_ 15:85) using the four-step vacuum swing adsorption (VSA) method under dry conditions [25]. An excellent percentage of CO_2_ purity (95%) and recovery (90%) was achieved using CALF-20. Although CALF-20 can be considered a promising scalable MOF for CO_2_ adsorption and separation, its capability for the adsorption of other toxic gases has not yet been established. Since the CALF-20′s framework is composed of abundant nitrogen and oxygen sites, comparing the adsorption of polar and non-polar gas molecules on the CALF-20 pore surface will be imperative as the surface polarity is a determinative agent for gas adsorption on the pore surface.

In this work, we have selected hydrogen sulfide (H_2_S), sulfur dioxide (SO_2_), fluorine (F_2_), and chlorine (Cl_2_) as polar and non-polar gas molecules to study. Adsorption of these gases is important in terms of the environmental and health aspects since they are among the most toxic gases with a high level of immediately dangerous to life or health (IDLH) status [26,27]. These gases are mainly released from the combustion of natural gas, biogas, and petroleum, which cause acute environmental and health problems. Halogen contaminations have been observed in some areas depending on industrial activities; for instance, a high level of fluorine pollution was found surrounding a power station in New South Wales (Australia) [28,29]. Studies have demonstrated that a sufficient concentration of H_2_S (e.g., above 100 ppm) damages the central nervous system in the human body [30,31]. In addition, the exposure of asthmatic people to H_2_S leads to bronchial constrictions [32,33]. The emission of SO_2_ in the air accelerates the formation of acid rain and photochemical smog [34]. Research works on the adsorption of SO_2_ using MOFs [35], zeolites [36], and ionic liquids [37] have attributed to the highly corrosive nature of SO_2_ gas, which can affect the materials. MOFs have been found to be the most widely used platform among them. [38,39,40]. Herein, two computational approaches, including grand canonical Monte Carlo (GCMC) and molecular dynamics (MD) simulations, were employed to study the adsorption and the mobility of the gas molecules, respectively. The details of the methods employed in this work are described in the next section. These methodologies offer a detailed, atomic-level understanding of the adsorption of both polar and non-polar gas species in CALF-20.

## 2. Computational Details

### 2.1. Model Construction

Crystal information file (.cif) was taken from Cambridge Crystallographic Data Center (CCDC) with deposition number 2084733 [22]. The .cif file was employed to perform initial geometry-based analysis, such as pore diameters using the Zeo++ program version 0.3 (http://www.zeoplusplus.org/, accessed on 28 December 2022) [41] and surface area using RASPA 2.0 program (https://github.com/iraspa/raspa2, accessed on 28 December 2022) [42]. The molecular formula of CALF-20 is [Zn_2_(1,2,4-triazolate)_2_(oxalate)], and the metal centers, zinc (Zn), are fully coordinated. The unit cell lengths of a, b, and c for CALF-20 are 8.91, 9.69, and 9.48 Å, respectively, and the arrangement of the atoms in the unit cell formed a monoclinic symmetry. The CALF-20′s unit cell was then extended to a 3 × 3 × 3 supercell. Periodic boundary conditions were defined in all directions for all simulations. Lennard-Jones (LJ) and electrostatic potentials were applied uniformly to CALF-20 framework atoms, in which the LJ potential parameters were described by GenericMOFs force field (as implemented in RASPA [42]) (see Supplementary Materials (Figures S1 and S2, Tables S1 and S2)). GenericMOFs is a combined force field where the metal center (Zn) is treated by the universal force field (UFF) [43] and the non-metallic elements by Dreiding force field [44]. Atomic charges of the frameworks were calculated using the CHarges from Electrostatic Potentials using a Grid (CHelpG) method at B3LYP/6-311+G(d,p) level (Figures S1 and S2, Tables S1 and S2). Atomic van der Waals radii used for Zn, O, N, C, and H were 1.39, 1.52, 1.55, 1.70 and 1.09 Å, respectively [45,46,47]. Lorentz-Berthelot mixing rules were employed to compute the adsorbent-adsorbate and adsorbate-adsorbate interactions. The structure of gas molecules was initially created and optimized at B3LYP/6-311+G(d,p) level, and the atomic charges were extracted using the electrostatic potential (ESP) method (Figure S3, Table S3). Afterward, all gas molecules were parameterized using the general amber force field (GAFF) (Figure S3, Table S3). Quantum mechanics (QM) calculations of atomic charges were performed using the Gaussian 09 program [48], whereas GCMC and MD simulations were carried out using RASPA 2.0 [42].

### 2.2. GCMC Simulation

**Figure 2:**
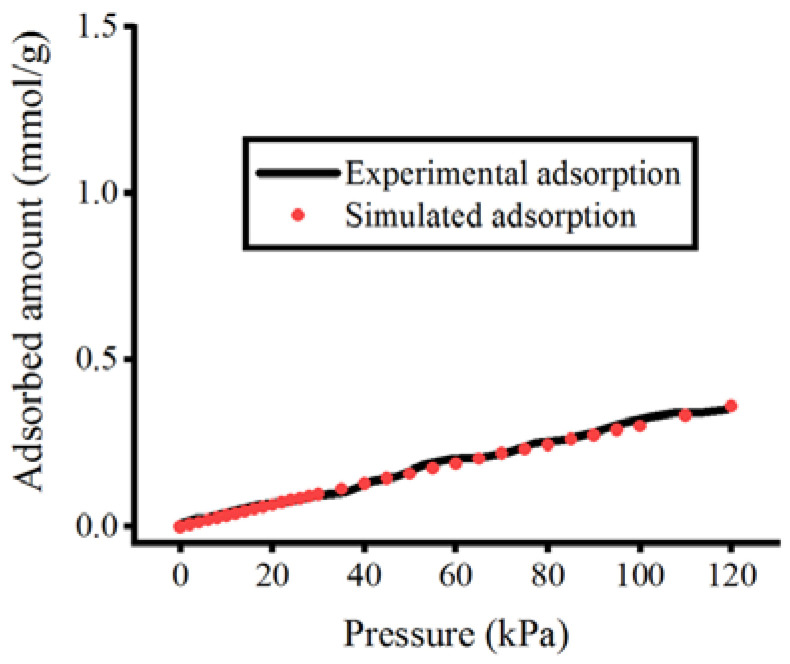
Comparison of experimental [22] and simulated adsorption isotherms of N_2_ in CALF-20.

To evaluate the adsorption isotherms of pure F_2_, Cl_2_, H_2_S, and SO_2_ gases at low-pressure, the GCMC method was employed. All the simulations were performed at 293 K [49] and a range of pressure between 0 to 120 kPa. Evaluating the adsorption properties in this condition of low pressure and ambient temperature is highly important. It allows us to characterize the parameters which control the adsorbed gas affinity, such as adsorbent-adsorbate interactions, surface analysis, and porosity [50,51]. The helium (He) void fraction and accessible pore volume of CALF-20 were determined to be 0.35 and 0.22 cm^3^/g, respectively. Calculation of the He void fraction for CALF-20 was performed using Widom particle insertion, and the resulting value (0.35) was set in the input file for adsorption simulations. Our calculated helium void fraction was comparable with the experimental value of 0.38 [22]. Up to 2.5 × 10^5^ Monte Carlo cycles were set for the adsorption simulations, which consisted of 5 × 10^4^ cycles for the initial equilibrium phase and 2 × 10^5^ cycles for the production phase. This number of cycles was sufficient for initial equilibration and production stages as used in similar works [52,53,54,55]. Each MC sampling move was set by the equal probability of attempting insertions, deletions, rotations, and translations [56]. In addition, as shown in Figures S4 and S5, the average potential energy and the number of adsorbed gases of the equilibrium phases at saturation pressure confirmed that the systems were adequately equilibrated. Each Monte Carlo (MC) move includes an equal probability of translation, rotation, insertion, and deletion. A 12.0 Å cut-off was defined in the RASPA package for the electrostatic and van der Waals interactions. To ensure the accuracy of our modeling for the framework and gas molecules, the adsorption isotherm of nitrogen (N_2_) gas in CALF-20 was validated with experimental values [22]. Similar to the gases used in this study, the N_2_ molecule was optimized at B3LYP/6-311+G(d,p) level, and the atomic charges were assigned using the ESP method. The topology for the N_2_ molecule was also parameterized using the GAFF force field. As depicted in Figure 2, our simulated adsorption isotherm ranging from 0 to 120 kPa is in good agreement with the experimental adsorption isotherm. 

### 2.3. MD Simulation

MD simulations were used to characterize the movement of both polar and non-polar gases within the pores of CALF-20. An equal concentration was adopted for all the gas molecules as 1 mole per unit cell, resulting in a total of 27 moles in the 3 × 3 × 3 frameworks. The distribution of 1-mole gas per unit cell, which means loading at very low pressure, would help us to understand the impact of the CALF-20 pore surface on the gas molecule’s motion (adsorbent-adsorbate) regardless of dominant adsorbate-adsorbate interactions [57,58]. MD simulations were performed at 293 K in a canonical (NVT) ensemble using the Nose-Hoover thermostat. The cut-off was defined as 12 Å for the electrostatic and the van der Waals interactions. Ewald method with a precision of 10^−6^ was set for computing the long-range electrostatic interactions. A full MD run was comprised of 10^7^ production cycles after accomplishing 10^4^ initialization cycles and 10^4^ equilibration cycles. The time step was set for 0.5 fs, and this resulted in a total of 5 ns of production simulation time. For our particular purpose, the total production MD simulation time is sufficient to generate the slope of mean square displacement (MSD) for gas molecules in porous materials [59,60,61,62]. However, to sample a higher frequency of MSD data during the simulation run, the order-N algorithm with 100 block elements was adopted.

## 3. Results and Discussion

Many attempts have been made to enlarge the pore size of MOFs with the purpose of greater gas storage [63]. So far, the pore sizes of MOFs have varied from 3 to 100 Å, and the surface area has ranged from 100 to 10,000 m^2^/g [64]. However, increasing the capability of MOFs for specific and selective gas adsorption required the pore size to be fine-tuned [65]. Fine-tuning MOF pores either by functionalizing the frameworks or selecting a shorter organic linker is beneficial because; (i) they promote the pore’s local charge density, thus making better adsorption at low pressure [66,67], and (ii) they discriminate gas molecules based on their three-dimensional molecular size [68,69]. Evaluation of pore diameter for CALF-20 using Zeo++ showed the largest cavity diameter (LCD) and the pore-limiting diameter (PLD) of 4.3 and 2.8 Å, respectively. The calculated N_2_ surface area using RASPA was determined to be 317 m^2^/g at 77 K, which is comparable to the experimental value of 528 m^2^/g at the same temperature. [22]. The two results from pore diameter and surface area gave us the expectation that CALF-20 has small enough (fine-tuned) pores for the selective adsorption of some toxic gases.

### 3.1. Adsorption Isotherm

**Figure 3:**
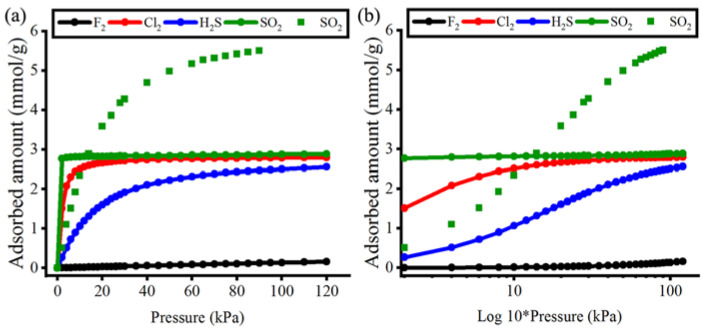
Simulated adsorption isotherms of gas molecules in CALF-20 MOF: (**a**) linear plot and (**b**) log10 plot. The square symbols represent the adsorption isotherms of SO_2_ in ZIF-69 [72].

The adsorption isotherms of polar (H_2_S and SO_2_) and non-polar (F_2_ and Cl_2_) gas molecules in CALF-20 at 293 K are illustrated in Figure 3. The isotherm plots are displayed using a logarithmic scale (log10) on the x-axis for clarification of the adsorptions at very low pressure (Figure 3b). The MOF pores were abruptly filled by SO_2_ and Cl_2_ at nearly zero pressure (near 0 kPa). This corresponds to the larger van der Waals surface of SO_2_ and Cl_2_ making greater contact with the MOF pore surface, resulting in higher adsorption. It is also noticeable that SO_2_ and Cl_2_ plots showed stable progression after about 20 kPa (green and red lines). The plateau state became more evident for SO_2_ at very low pressure after the log10 plot was drawn. These types of adsorption (having a constant isotherm) indicate that the MOF pores are completely saturated [70,71]. Shimizu et al. [22] acquired high storage of CO_2_ in CALF-20 at 120 kPa (up to 5.0 mmol/g at 273 K), whereas we have highlighted that CALF-20 has abrupt and selective adsorption for SO_2_ molecules at 293 K and nearly zero pressure. The difference implies that CO_2_ adsorption using CALF-20 is dependent on higher pressure (more energy-consuming), whereas SO_2_ adsorption relies on better fitting into the MOF pores, implying adequate molecular size and geometry. We additionally compare the adsorption isotherms of SO_2_ in CALF-20 to another framework, ZIF-69 [72]. ZIF-69 pore surface has a similar chemical environment to the CALF-20 pore surface since it is composed of nitrogen-containing rings, four fully coordinated Zn metals, and nitro functional groups. However, ZIF-69 was found to have a larger pore diameter, with an LCD of 8.7 Å and a PLD of 4.9 Å [73], nearly double the values observed in CALF-20. Adsorption isotherms of SO_2_ in ZIF-69 slightly increased at very low pressure meaning that ZIF-69 had enough empty pore space for higher loading of SO_2_ gas while the pressure was increasing. The adsorption isotherm for SO_2_ in ZIF-69 also showed that it reached a saturation loading that was nearly twice as high as that of SO_2_ in CALF-20, highlighting the difference in pore sizes between the two materials. By this comparison, CALF-20 has again demonstrated a fine-tuned pore size for selective adsorption of SO_2_ gas.

Meanwhile, F_2,_ with the smallest molecular size, made the lowest amount of loading inside CALF-20 pores. The very slow growth of F_2_ adsorption at low pressures suggested that CALF-20 should not be used to adsorb and store F_2_ gas. H_2_S showed a gradually increased loading suggesting that H_2_S molecules were adsorbed on the MOF pore surfaces and then accumulated in the pores as the pressure increased [74]. At the end of the simulations, the maximum loading of the gas molecules at 120 kPa was observed in decreasing order of SO_2_ (2.89 mmol/g) > Cl_2_ (2.79 mmol/g) > H_2_S (2.55 mmol/g) > F_2_ (0.16 mmol/g).

### 3.2. Radial Distribution Function (RDF)

To study the structural behavior of the different gas species in the system, the radial distribution function (RDF) was calculated using Equation (1).

(1) $$g_{\mathrm{ij}}\left(r\right)=\frac{N_{\mathrm{ij}}\;\left(r,\;r\;+\Delta r\right)\cdot V\;}{4{\pi r}^{2}\cdot \Delta r\cdot N_{i\;}\cdot N_{j}\;}$$

**Figure 4:**
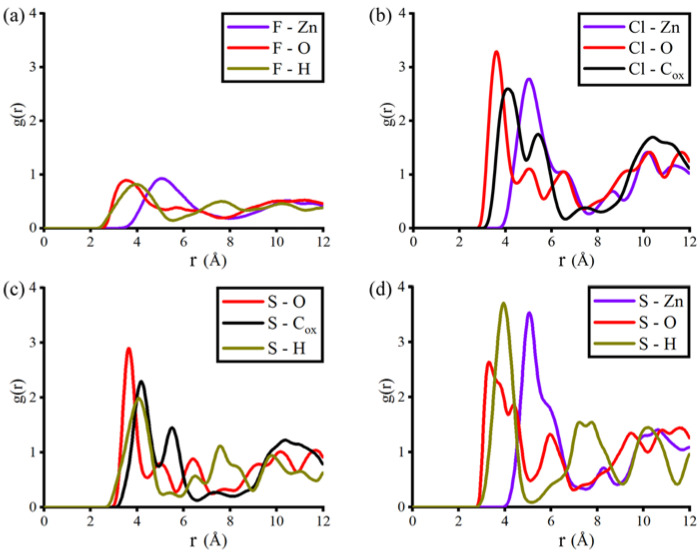
Radial distribution plots for (**a**) F_2_, (**b**) Cl_2_, (**c**) H_2_S, and (**d**) SO_2_ gases in CALF-20.

In this equation, N_ij_ (r, r + Δr) represents the number of particles j around particle i inside the area from r to r + Δr. V and N denote the volume of the system and the number of particles, respectively. Analyzing RDF allowed us to assess the distribution of F_2_, Cl_2_, H_2_S, and SO_2_ gas molecules on the interaction sites of CALF-20. In general, the highest distribution of gas molecules must follow the adsorbed amount in the CALF-20 pores. The level of distribution for the gas molecules was found in the order of F_2_ < H_2_S < Cl_2_ < SO_2_ (Figure 4). Since F_2_ had the least adsorption and more freely moving inside CALF-20 pores, the RDF calculation produced the lowest distribution of this molecule (about ~0.9 g(r)). Zinc and oxygen atoms in the frameworks were found to be the most favorable sites for F_2_ molecules. In contrast, the distribution g(r) for sulfur atoms in SO_2_ molecules reached the highest level (about ~3.7 g(r)) when they had interactions with zinc and hydrogen atoms of the framework.

Among the interaction sites in CALF-20, the oxygen atom was the most active site, as it formed the strongest interaction (the shortest distance of distribution) with the gas molecules. This oxygen atom belonged to the oxalate ions, which built a strong coordinative bond with zinc during the later step in the synthesis of CALF-20 (Figure 1). According to the adsorbed amounts and the van der Waals surfaces of F_2_, Cl_2,_ and H_2_S, the RDF plots for these gas molecules exhibited a reasonable distribution of oxygen atoms in CALF-20. However, an abnormal RDF plot was seen for the sulfur atom of SO_2_ gas on oxygen atoms in the framework (Figure 4d). This phenomenon limited the availability of the sulfur atom to interact with oxygen atoms in the framework, which encouraged us to carry out further analysis of adsorbent-adsorbate and adsorbate-adsorbate interaction energies.

### 3.3. Simulation Snapshot Analysis

**Figure 5:**
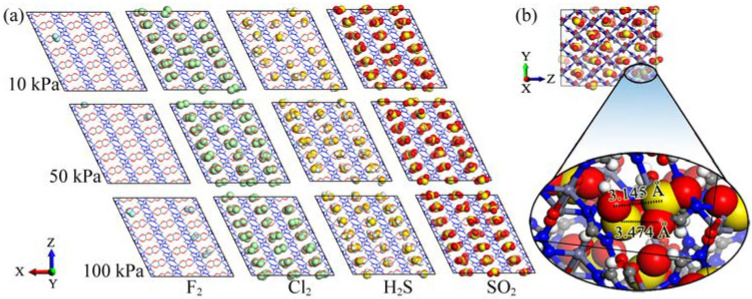
(**a**) Simulation snapshots of F_2_, Cl_2_, H_2_S, and SO_2_ adsorption in CALF-20 framework at 10, 50, and 100 kPa as visualized along the Y-axis, (**b**) magnified SO_2_ packing in CALF-20 pores, presented along X-axis of the framework.

The simulation snapshots for the adsorption of the gas molecules F_2_, Cl_2_, H_2_S, and SO_2_ at 10, 50, and 100 kPa along the Y-axis of CALF-20 are presented in Figure 5a. As illustrated, all gas molecules were contained between the layers of 1,2,4-triazolate-bridged zinc(II). These furrows between the layers are shelved by oxalate ions, which create the active sites for gas molecule adsorption. F_2_ gas exhibited poor loading between triazolate sheets because of its small van der Waals surface, thus, the least contact with the surface of the pores. It could be seen that the increase in pressure had no significant impact on the adsorption of F_2_. The framework was also gradually loaded by H_2_S molecules on oxalate sites by increasing the pressure. In contrast, CALF-20 channels along the *y*-axis were almost saturated by the accumulation of Cl_2_ and SO_2_ gas molecules from 10 to 50 and 100 kPa. By closer exploration of the three-dimensional SO_2_ packing into CALF-20 pores along the *x*-axis, it was realized that SO_2_ molecules were able to construct gas packing through SO-O···SO_2_ intermolecular interactions since they have angular and polar structure (Figure 5b). The SO_2_ intermolecular interaction distances between oxygen and sulfur atoms (OSO···SO_2_) were found to be 3.145 and 3.474 Å in CALF-20 pores, which indicated good agreement with experimental high-resolution SPXRD measurement at 100 kPa and 293 K [75]. Intermolecular interactions of SO_2_ can be considered as evidence explaining the reduced distribution of sulfur atoms in SO_2_ molecules on oxygen atoms in the CALF-20 framework (Figure 4d). Nevertheless, this situation was not found for the other types of gases (F_2_, Cl_2,_ and H_2_S). To show the effect of intermolecular interactions on the adsorption of all gas molecules in CALF-20, an analysis of interaction energies was carried out.

### 3.4. Interaction Energies

**Figure 6:**
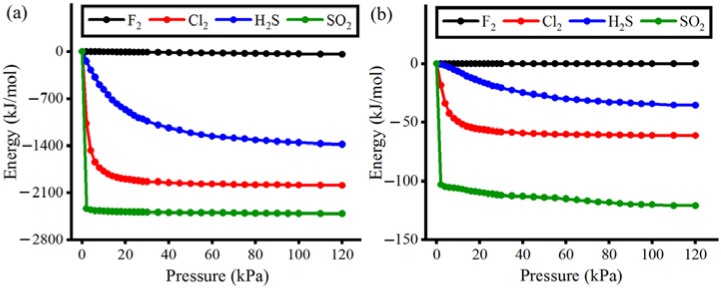
Interaction energy for (**a**) adsorbent-adsorbate and (**b**) adsorbate-adsorbate.

Figure 6 shows the adsorbent-adsorbate and adsorbate-adsorbate interaction energies at pressures ranging from 0 to 120 kPa at 293 K. The interaction energy was obtained by the sum of van der Waals interactions and Columbic interactions that are governed by atomic charges [76]. According to the plot in Figure 6a, framework-F_2_ and framework-SO_2_ interaction energies remained constant throughout, whereas the framework-H_2_S and framework-Cl_2_ interaction energies steadily reduced at very low pressure and reached a plateau at a higher pressure. The framework-F_2_ showed the highest values of interaction energies because the F_2_ molecule had the least van der Waals surface for the interaction with the framework surface, and the atomic charge was neutralized (no Columbic interactions). On the other hand, the framework-SO_2_ interaction energies were the lowest values, around −2300 kJ/mol, since the SO_2_ molecule provided the largest van der Waals surface and the greatest polarity (the strongest columbic interactions). The highest polarity in SO_2_ is the consequence of uneven charge distribution between oxygen and sulfur atoms (see Table S3 in Supplementary Materials for the values of atomic charges for all gas molecules). These characteristics in the SO_2_ molecules allowed them to have the strongest chemical affinity and interaction with the surface of CALF-20 pores.

This trend at very low pressure indicates that H_2_S and Cl_2_ molecules were not optimally fitted within the MOF pores (lower chemical affinity) and did not make strong interactions with the surface of the pores as compared to SO_2_ molecules. Although similar trends were observed for adsorbent-adsorbate and adsorbate-adsorbate interaction energies, we realized an interesting point in Figure 6b. When comparing the adsorbate-adsorbate interaction energies, the energy level of F_2_-F_2_, Cl_2_-Cl_2,_ and H_2_S-H_2_S intermolecular interactions have consistent gaps. However, the difference in energy level between Cl_2_-Cl_2_ and SO_2_-SO_2_ intermolecular interactions is almost doubled. Regarding their molecular structure, Cl_2_ is linear and non-polar, but SO_2_ is an angular and highly polar molecule that can make tightly packed molecules. The tighter SO_2_ packing was governed by a considerable difference between the positively charged sulfur atom (+0.62 e) and the negatively charged oxygen atom (−0.31 e) of other SO_2_ molecules. The angular shape and charge differences between sulfur and oxygen atoms in SO_2_ led to a stronger attraction via electrostatic interactions among SO_2_ gas molecules. Therefore, we are convinced that SO_2_ gas molecules not only built the strongest adsorbent-adsorbate interactions but also had a much tighter gas packing inside CALF-20 pores. 

### 3.5. Henry Coefficient (K_H_) and Isosteric Heat of Adsorption (Q_st_)

The Henry coefficient (*K*_H_) and the isosteric heat of adsorption (*Q*_st_) are useful metrics to measure the strength of chemical affinity of guest molecules in MOFs pores [77,78]. In this study, the *K*_H_ value for each gas@CALF-20 complex system was calculated using the Widom test particle insertion method at very low pressure, and the results were compiled in Table 1. The *K*_H_ value can also be used for evaluating the selectivity when it is extracted at low pressure and low concentration [79,80]. The values of *K*_H_ for the corresponding gas molecules in CALF-20 follow the order of F_2_ > H_2_S > Cl_2_ > SO_2_. These results conveyed that SO_2_ molecules were adsorbed on CALF-20′s pore surfaces more than 20 times stronger than Cl_2_ (22.743/1.018). Using the *K*_H_ values for each gas species, the separation ability of CALF-20 for binary gas mixture can be assessed using intrinsic thermodynamic selectivity (*α*) [81,82]. The *α* values for SO_2_/H_2_S, SO_2_/Cl_2_, SO_2_/F_2_, Cl_2_/H_2_S, H_2_S/F_2_, and Cl_2_/F_2_ mixtures were measured as 163.62, 22.34, 22,743, 7.32, 139, and 1018, respectively. Thus, it is predicted that CALF-20 is highly promising for the separation of SO_2_/F_2_ and Cl_2_/F_2_ mixtures, contrarily not recommended for Cl_2_/H_2_S and SO_2_/Cl_2_ mixtures.

The *Q*_st_ value is an indication of the strength of the interactions between the frameworks and the gas molecules. The higher value of *Q*_st_ means stronger interaction between the two components. The *Q*_st_ value can be calculated using Equation (2),

(2) $$Q_{\mathrm{st}}=\;\mathrm{RT}-{\left(\frac{\partial U}{\partial N}\right)}_{T,V}$$

where the *∂*U/*∂*N is calculated as average over configurations; R, T, U, and N are the ideal gas constant, temperature, total energy of the system, and the number of adsorbed molecules, respectively. Although there are many studies in the literature that have reported high values of *Q*_st_ for the adsorbed gas in different materials [83], we are interested in highlighting the values of *Q*_st_ for CALF-20 against porous metallocavitand pillarplex (PPX) and a covalent organic framework (COF), COF-10. PPX is formed by pyrazole rings where connected to Gold (Au) metal. The presence of nitrogen atoms in the pyrazole rings of PPX pores provides polar sites, similar to triazole rings in CALF-20. In addition, the cavity diameter in PPX has been reported as 4.3 Å [83], which is equivalent to the LCD in CALF-20 measured by the Zeo++ program. Adsorptions of a wide variety of gas molecules inside the PPX pore were previously studied by computational tools [84]. According to the adsorption isotherms and the *Q*_st_, CS_2_, H_2_S, NO_2_, HBr, and Br_2_ were selected as the highest selective adsorption because of the greatest value of *Q*_st_ among all gas molecules. Surface area and pore diameter were found to be considerably high in COF-10 (1760 m^2^/g and 32 Å, respectively) [85] compared to our observation for CALF-20 (317 m^2^/g and 4.3 Å, respectively). In this regard, Zeng et al. concluded that COF-1 had the highest uptake of H_2_S and SO_2_ compared to COF-5, COF-8, and COF-6 due to its largest surface area and pore volume [86]. However, COF-10 had smaller *Q*_st_ values caused by the differences in the COF’s surface and structure. In addition, the authors stated that COFs such as COF-10 having too large a pore could not be appropriate for high selective adsorption of H_2_S and SO_2_ at low pressure. Based on Table 1, the *Q*_st_ values of CALF-20 indicate that it was capable of adsorption for polar gas molecules due to having a polar surface and capture of non-polar gas molecules by providing small pore volume (high chemical affinity). Therefore, CALF-20 could be considered a highly promising material for separating polar and non-polar molecules. The polarity of a gas molecule is an advantage for higher adsorption due to stronger adsorbate-adsorbate interactions.

When the value of *Q*_st_ is less than 41.84 kJ/mol, the adsorption forms via physical adsorption (physisorption) [87]; the phenomenon of adsorption of gas molecules (adsorbates) on solid materials (adsorbents) goes through two main mechanisms; chemisorption and physisorption [88]. Chemisorption is accomplished when adsorbates form strong chemical bonds to the active sites of the adsorbent surface, resulting in a unimolecular layer of adsorbates. However, physisorption takes place when adsorbates build weak van der Waals forces on the adsorbent surfaces, resulting in a multilayer of adsorbates. According to Table 1, F_2_ and H_2_S gas molecules formed physisorption onto the CALF-20 pore surface. It was found that H_2_S was adsorbed more than F_2_ (Figure 3). It is expected that H_2_S molecules built greater multilayer by weak van der Waals forces. This result could be considered for addressing a common problem in the use of metal oxides such as MgO, Ni-doped MgO, and ZnO to remove and separate H_2_S as they are irreversible transformation materials due to the strong chemical interactions with H_2_S [89]. Interestingly, Cl_2_ in CALF-20 recorded a *Q*_st_ value on the border (41.84 kJ/mol), which gave the possibility of chemisorption. This could be attributed not only to the highly reactive nature of Cl_2_ [90] but also to fitting CALF-20 pore size to Cl_2_ molecules.

Concerning the possibility of chemisorption of Cl_2_ in CALF-20, Dinca and colleagues proved that Cl_2_ and Br_2_ gases made strong chemical bonds on the Co metal sites of MOF-74 [91]. However, the reactions between the Co metals and the halogen gases were reversible as thermal treatment of the MOF led to the breaking of the Co-halogen bonds. Adsorption of SO_2_ in CALF-20 went through chemisorption, and it probably happened on Zn metal sites, as demonstrated by RDF analysis (Section 3.2). In comparison, in many other frameworks that are constructed by fully coordinated Zn metals, such as MOF-5 [92] and ZIFs [93,94,95], SO_2_ mostly tends to be physisorbed on the pore surfaces. For example, Yazaydin et al. calculated the values of *Q*_st_ for SO_2_ loaded in ZIF-10, ZIF-68, ZIF-69, and ZIF-71 as 26.1, 52.6, 36.7 and 26.0 kJ/mol, respectively, at low pressure and 298 K [72]. As a result, only ZIF-68 was able to provide chemisorption for SO_2_ due to its appropriate pore size and the presence of -NO_2_ functional groups. It is important to note that materials such as ZIF-68 and CALF-20, which are suitable platforms for strong adsorption (chemisorption), can be effectively utilized for capturing SO_2_.

### 3.6. Mean Square Displacement (MSD)

MD simulations were carried out to elucidate the mobility of the gas molecules in CALF-20 pores [96,97]. All MD simulations were conducted at 293 K in the NVT ensemble, and the resulting trajectories were used to extract the mean square displacement (MSD) of the gas molecules. The values of MSD were computed via the Einstein relationship [98,99] as presented in Equation (3), and they allowed us to express the average distance traveled by the gas molecules.

(3) $$\mathrm{MSD}\;\left(t\right)=\frac{1}{N}\overset{N}{\underset{i=1}{\sum }}{\left|r_{i}\left(t\right)-{\;r}_{i}\left(0\right)\right|}^{2}$$

**Figure 7:**
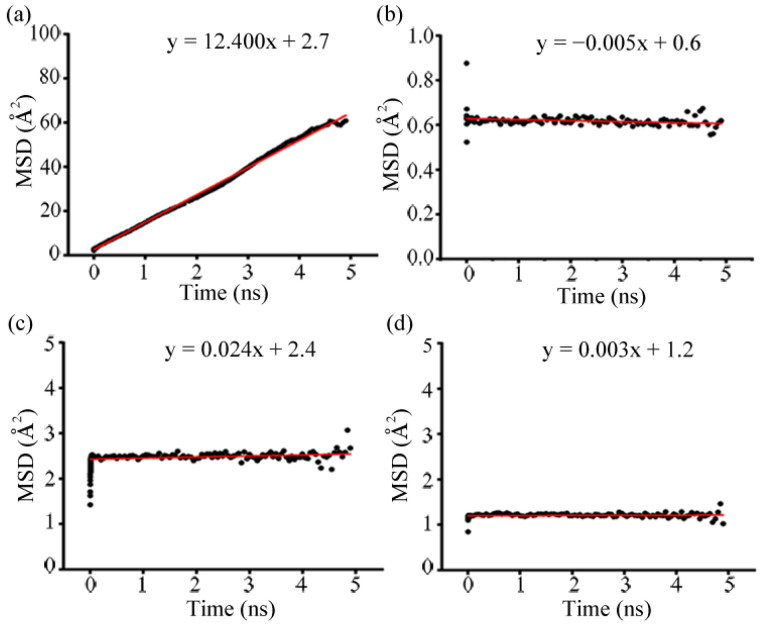
Mean square displacements for (**a**) F_2_, (**b**) Cl_2_, (**c**) H_2_S, and (**d**) SO_2_ in CALF-20 during 5 ns of MD simulations.

According to Equation (3), N, t, and r_i_ are the number of particles, time, and center-of-mass of the particle i, respectively. The MSD plots for one mole of F_2_, Cl_2_, H_2_S, and SO_2_ gas per unit cell of CALF-20 (a total of 27 moles in a 3 × 3 × 3 MOF system) are presented in Figure 7, and the calculated slope of MSD (red line) is correlated to the diffusion of the gas molecules. The results from MSD allowed us to measure the self-diffusion coefficient (*D*_s_) using Equation (4) [100]:

(4) $$D_{s}=\frac{1}{2d}\;\underset{\Delta t\to \infty }{\lim}\mathrm{MSD}\;\left(\Delta t\right)$$

where d is equal to 3 in the case of three-dimensional systems, the highest value of MSD slope belonged to F_2_ (12.400), and it suggested that F_2_ molecules were moving freely within the CALF-20 pores. Accordingly, the average area spent by F_2_ molecules was enhanced up to ~60 Å^2^. In contrast, the lowest values of the average area were found for Cl_2_ (~0.6 Å^2^) and SO_2_ (~1.0 Å^2^). The MSD slope for Cl_2_ and SO_2_ were recorded as −0.005 and 0.003 (almost zero), respectively. This indicates that Cl_2_ and SO_2_ gas had the slowest motion in CALF-20 pores, and they did not considerably change during the 5 ns simulation time. This can be attributed to the tightly fitted Cl_2_ molecules inside the pores and the strongest polar interactions of SO_2_. In the case of H_2_S, the average area value was about ~2.5 Å^2^ during the simulation time with a steadily increased MSD slope of 0.024. It meant that the mobility of H_2_S molecules in CALF-20 pores slowly progressed as the simulation time passed. The mobility of the gas molecules in the MOF pores was in the order of F_2_ > H_2_S > SO_2_ > Cl_2_. Considering the same amount of gas molecules in the framework, the values of self-diffusion coefficient (*D*_s_) were obtained as 2.7, −0.0008, 0.004, and 0.0005 Å^2^ ns^−1^ for F_2_, Cl_2_, H_2_S, and SO_2_, respectively. These values stated that diffusion of F_2_ gas molecules inside the CALF-20 pores took the largest space at very low pressure, whereas the rest of the gases had much-restricted motion in their position.

## 4. Conclusions

In this computational study, we have presented an atomic-level understanding of the adsorption of polar (H_2_S and SO_2_) and non-polar (F_2_ and Cl_2_) toxic gases in CALF-20 at low pressure. GCMC and MD methods were employed to calculate the adsorption isotherms, radial distribution functions, mean square displacements, Henry coefficients, and heat of adsorptions for different systems. The adsorption isotherm showed the highest and constant loading for SO_2_ and Cl_2_ gas at low pressure. Gas molecules having larger contact with the framework surface (e.g., SO_2_ and Cl_2_) have higher adsorption, with polar molecules such as SO_2_ showing much higher adsorption due to the strongest adsorbent-adsorbate interactions. This was further confirmed by the highest amount of heat of adsorption obtained for SO_2_ (45.51 kJ/mol). RDF analysis elucidated that oxygen atoms of the framework belonging to the oxalate component are the most favorable interaction site with all gas species tested. As measured by MSD analysis, smaller and non-polar gas molecules such as F_2_ (MSD = 12.4 Å^2^) could not be loaded sufficiently in CALF-20 at low pressure. The adsorption of F_2_ in CALF-20 was very poor, and the molecules were freely moving in pores since they lacked charge and had not had enough contact surfaces with the framework. Based on the results, we have determined CALF-20 as a highly potential material for selective adsorption of SO_2_ and Cl_2_ at low pressure.

## Figures and Tables

**Table 1:** The values for Henry coefficient (*K*_H_) and isosteric heat of adsorption (*Q*_st_).

Gas	*K*_H_ CALF-20	*Q*_st_ PPX ^1^	*Q*_st_ COF-10 ^2^	*Q*_st_ CALF-20 ^3^
F_2_	0.001	11.72	-	16.35
Cl_2_	1.018	35.15	-	41.84
H_2_S	0.139	28.45	15.75	31.88
SO_2_	22.743	-	17.68	45.51

*K*_H_: mol/kg/kPa and *Q*_st_: kJ/mol; ^1^ 100 kPa 298 K, ^2^ 100 kPa 303 K, ^3^ 100 kPa 293 K.

## Data Availability

The datasets generated during the current study are available from the corresponding author at a reasonable request.

## Supplementary Materials

All materials are included in the Supplementary Materials file, available online at https://www.mdpi.com/article/10.3390/polym15030760/s1, Figure S1: The first cluster used for CHelpG charges calculation; Figure S2: The second cluster used for CHelpG charges calculation; Figure S3: Optimized structure of the gas molecules; Figure S4: Plots for potential energy of the systems; Figure S5: Plots for number adsorbed gases; Table S1: The values for the atomic charges and the LJ potential parameters; Table S2: The values for the atomic charges and the LJ potential parameters; Table S3: The values for the atomic charges and the LJ potential parameters.
