# Supplementary material for: Exploring the Potential of a Highly Scalable Metal-Organic Framework CALF-20 for Selective Gas Adsorption at Low Pressure
Source: Polymers (Basel). 2023 Feb 2;15(3):760. doi: 10.3390/polym15030760 (PMC9921038; doi:10.3390/polym15030760)
Supplement: Supplementary file 1 [file polymers-15-00760-s001.zip › polymers-2159376-supplementary.pdf]

Supplementary Materials

Exploring the Potential of a Highly Scalable Metal-Organic Framework CALF-20 for Selective Gas Adsorption at Low Pressure

Mostafa Yousefzadeh Borzehandani [1,2], Majid Namayandeh Jorabchi [3,*], Emilia Abdulmalek [1], Mohd Basyaruddin Abdul Rahman [1,2] and Muhammad Alif Mohammad Latif [1,2,4,*]

[1] Integrated Chemical BioPhysics Research, Faculty of Science, Universiti Putra Malaysia, 43400 Serdang, Selangor, Malaysia
[2] Foundry of Reticular Materials for Sustainability, Institute of Nanoscience and Nanotechnology, Universiti Putra Malaysia, 43400 Serdang, Selangor, Malaysia
[3] Leibniz Institute for Catalysis, Albert-Einstein-Straße 29a, D-18059 Rostock, Germany
[4] Centre of Foundation Studies for Agricultural Science, Universiti Putra Malaysia, 43400 Serdang, Selangor, Malaysia
* Correspondence: majid.jorabchi@catalysis.de (M.N.J.); aliflatif@upm.edu.my (M.A.M.L.)

Contents

I. Lennard-Jones (LJ) potential parameters and atomic charges for CALF-20 ……..........…................... S2
II. Lennard-Jones (LJ) potential parameters and atomic charges for gases ……………………..….…..... S3
III. Potential energy …………………………………..…………………………………..………..……..... S4
IV. Number adsorbed gases ………………………………….……………………………….………..……..... S4

I. Lennard-Jones (LJ) potentials and atomic charges for CALF-20

The cluster structures used for calculation of atomic charges are presented in Figure S1 and Figure S2. The first structure was used to extract the values of atomic charge for oxalate component and zinc (Zn). Also, the second structure was separately considered to calculate the atomic charge of triazolate ring. This is because each first and second cluster structure has good representative for oxalate and triazolate components, respectively. Subsequently, atomic charges of each structure was obtained by using the CHarges from Electrostatic Potentials using a Grid (CHelpG) method at B3LYP/6-311+G(d,p) level. Moreover, the LJ potential parameters for the corresponding atoms based on GenericMOFs force field are compiled in S1 and S2.

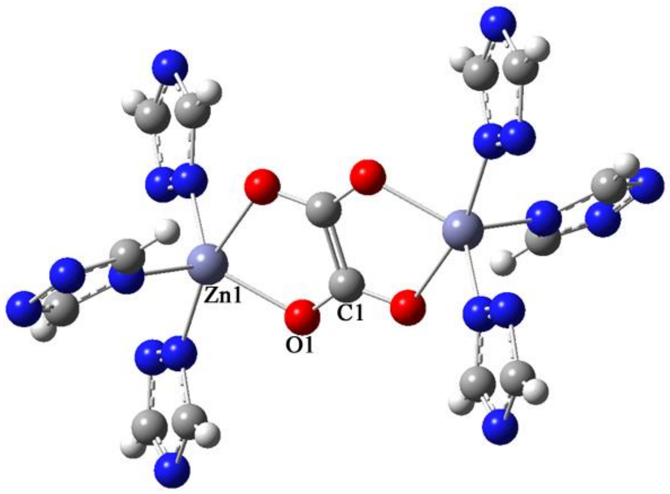

Figure S1. The first cluster used for CHelpG charges calculation.

Table S1. The values for the atomic charges and the LJ potential parameters.

Atom	Zn1	O1	C1
**Charge (e)**	0.658491	-0.551410	0.594723
**ε (K)**	62.3992	48.1581	47.8562
**σ (Å)**	2.46155	3.03315	3.47299

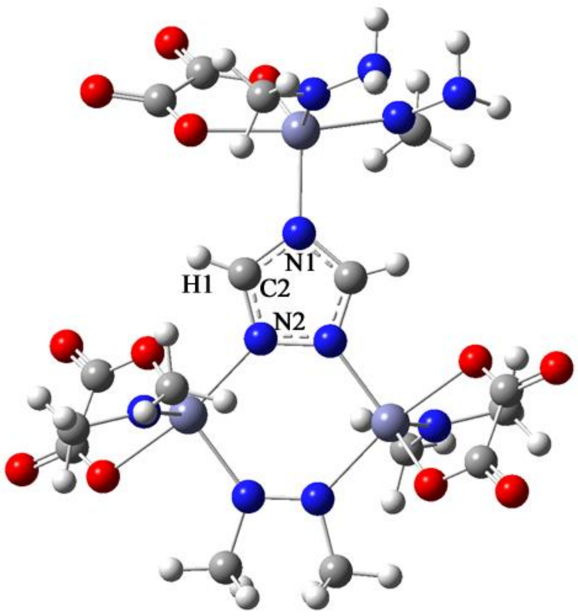

Figure S2. The second cluster used for CHelpG charges calculation.

Table S2. The values for the atomic charges and the LJ potential parameters.

Atom	C2	H1	N1	N2
**Charge (e)**	0.150849	0.128291	-0.226905	-0.232464
**ε (K)**	47.8562	7.64893	38.9492	38.9492
**σ (Å)**	3.47299	2.84642	3.26256	3.26256

II. Lennard-Jones (LJ) potentials and atomic charges for gases

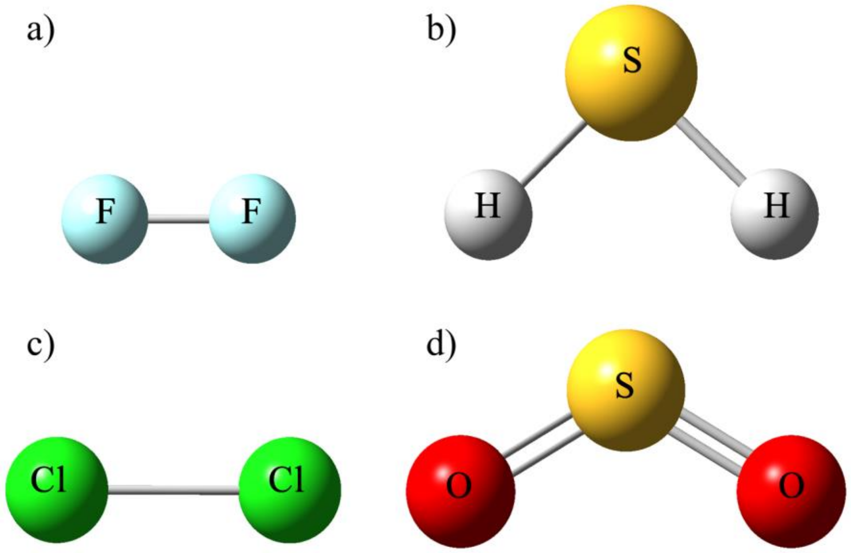

Figure S3. Optimized structure of the gas molecules.

Table S3. The values for the atomic charges and the LJ potential parameters.

**Gas**	**F_2**	Cl_2	H_2S		SO_2	
**Atom**	**F**	Cl	S	H	S	O
**Charge (e)**	0.00000	0.00000	-0.33500	0.16750	0.62178	-0.31089
**ε (K)**	41.8682	132.7502	142.10990	6.23996	142.10990	73.62146
**σ (Å)**	3.03422	3.46595	3.53241	1.08903	3.53241	3.04812

III. Potential energy

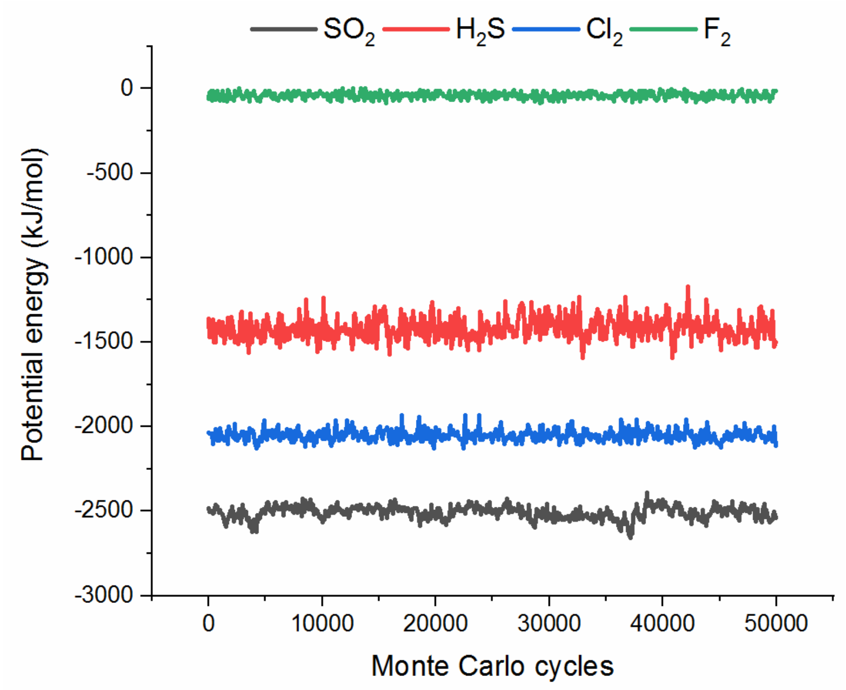

Figure S4. Plots for potential energy of the systems.

IV. Number adsorbed gases

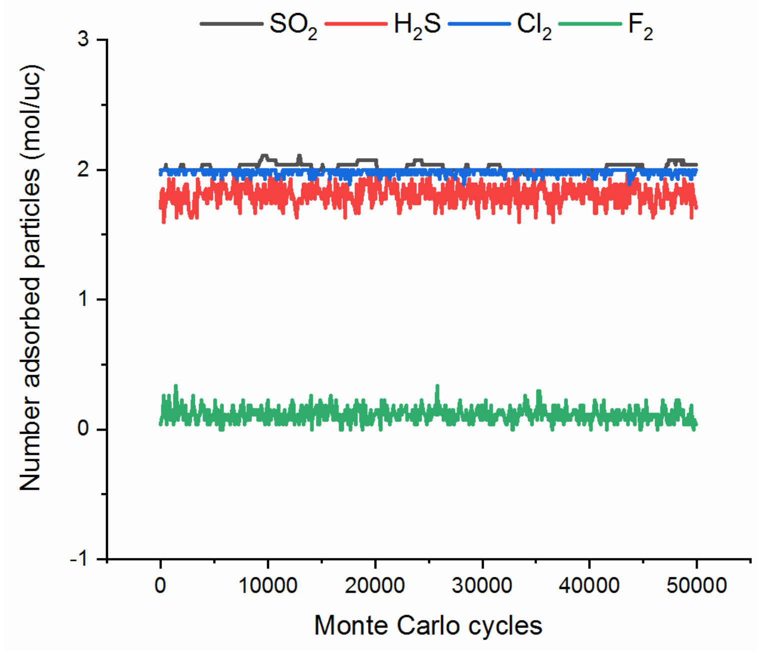

Figure S5. Plots for number adsorbed gases.
